# Exploring the Gut Microbiome and Metabolome in Individuals with Alopecia Areata Disease

**DOI:** 10.3390/nu16060858

**Published:** 2024-03-15

**Authors:** Olga Nikoloudaki, Daniela Pinto, Marta Acin Albiac, Giuseppe Celano, Alessio Da Ros, Maria De Angelis, Fabio Rinaldi, Marco Gobbetti, Raffaella Di Cagno

**Affiliations:** 1 Faculty of Agricultural, Environmental and Food Sciences, Free University of Bozen-Bolzano, 39100 Bolzano, Italymarco.gobbetti@unibz.it (M.G.); raffaella.dicagno@unibz.it (R.D.C.); 2 Human Microbiome Advanced Project (HMAP), Giuliani S.p.A, 20129 Milan, Italy; dpinto@giulianipharma.com (D.P.); fabio.rinaldi@studiorinaldi.com (F.R.); 3 Department of Soil, Plant and Food Science, University of Bari Aldo Moro, 70121 Bari, Italy; giuseppe.celano@uniba.it (G.C.); maria.deangelis@uniba.it (M.D.A.)

**Keywords:** alopecia areata, immunoregulation, gut microbiome, metabolome, biomarkers

## Abstract

In recent years, heightened attention has been devoted to unravelling the intricate interplay between genetic and environmental factors shaping the gut microbiota and its significance for human health. This study delves into exploring the plausible connection between Alopecia Areata (AA), an autoimmune disease, and the dynamics of the gut microbiome. Examining a cohort of healthy adults and individuals with AA, both the gut microbiota composition and volatile organic compound (VOC) metabolites from faeces and urine were analysed. While overall microbiota composition showed no significant differences, intra-individual variability revealed distinctions related to age, gender, and pathology status, with AA individuals exhibiting reduced species richness and evenness. Differential abundance analysis identified microbial biomarkers for AA, notably Firmicutes, *Lachnospirales*, and *Blautia*, while *Coprococcus* stood out for healthy individuals. The Data Integration Analysis for Biomarker discovery using Latent Components (DIABLO) method further supported these findings including metabolite biomarkers, such as esters of branched chain fatty acids and branched chain amino acids as predictors for AA, suggesting potential links to oxidative stress. Despite certain limitations, the study highlights the complexity of the gut microbiome and its metabolites in the context of AA, while the biomarkers identified could be useful starting points for upcoming studies.

## 1. Introduction

The human gastrointestinal (GI) tract harbours a complex and dynamic population of microorganisms, which is sparse in the stomach and upper intestine, but abundant in the colon tract [1]. The gut microbiome in its totality plays a key role in maintaining host homeostasis and health status [2,3,4]. Consciously, the modification of the gut microbiota and its related metabolome are intimately linked to numerous genetic, nutritional, and environmental determinants [5]. Nevertheless, the environment appears to be primarily responsible for shaping the human microbiota compared to host genetics [2]. For instance, the immune’s system abnormal generation of autoantibody-producing B cells and autoreactive T cells and the anomalous production of proinflammatory cytokines are some examples of the influence of environmental factors [6,7]. In this scenario, the association between alteration of the gut microbiome in patients suffering from autoimmune diseases such as inflammatory bowel disease (IBD) and multiple sclerosis (MS) has recently been investigated [8,9,10,11,12].

Among autoimmune disorders, Alopecia Areata (AA), originally called “Area Celsi”, is one of the most common forms of non-scarring alopecia, whose prognosis is still difficult and unpredictable [13,14,15]. Although the triggers of alopecia areata (AA) remain partially understood, it is widely acknowledged that the loss of immune privilege (IP), subsequent injury to hair follicle cells, and the promotion of inflammatory pathways are key factors considered prerequisites for the development of AA [16,17]. AA is considered a T-cell dependent autoimmune disease [18]. C8^+^ T cells are predominant in the follicular epithelium and are thought to be primarily responsible for the damage, while CD4^+^ lymphocytes aid the CD8^+^ response [19]. The loss of the normal IP of growing hair follicles in the active stage of the disease is associated with the occurrence of inflammatory cells, where the substance P (SP)-dependent modulation of IgM and interferon-γ (IFN-γ), a neuropeptide involved in pain transmission, are the key inducers of this phenomena [20,21]. The aetiology of AA presents a high activity of natural killer (NK) cells, and a dysregulated expression of the activation receptor NKG2D by activating NK receptors and its ligands, which are also implicated in other autoimmune diseases [22]. The imbalanced ratio of Th17 lymphocytes and T cells (Treg lymphocytes) is engaged in the pathophysiology of autoimmune conditions [23]. Hence, Th17 cells are involved in the pathogenesis of AA [24].

Human gut microbiome dysbiosis appears to also be a driving factor in other skin diseases such as atopic dermatitis (AD) and psoriasis [25,26]. Among diseases affecting the scalp, lichen planopilaris, a form of cicatricial alopecia, has been recently inspected in terms of microbiota composition and volatile compound profiles obtained by directly sampling scalp biopsy layers [27]. The onset and maintenance of the autoimmune skin diseases, including AA, is recognized as a possible cause of the depletion of bacteria that are beneficial for their anti-inflammatory properties including the release of short chain fatty acids (SCFAs) [28]. The intricate relationship between the gut and skin is bidirectional, with lifestyle factors like diet and hygiene significantly affecting immune tolerance and microbial dysbiosis. Notably, a Western diet and overly hygienic practices are linked to the development of immune-mediated inflammatory diseases, highlighting the importance of the gut–skin axis in understanding conditions like rheumatoid arthritis, psoriasis, and atopic dermatitis [25]. Despite the metagenomic evaluations of gut microbiota showing correlation with autoimmune disorders, at present, only few studies investigated the structure of gut microbiota in AA patients regardless of the possible interaction with gut metabolites [23,29,30].

In this study, we combined 16S rRNA gene sequencing and metabolomics to thoroughly characterize and compare faecal and urine samples from AA patients and healthy individuals (HI) using a novel integrative method (DIABLO) to identify multi-omics biomarkers that can discriminate between the phenotypic groups (AA vs. HI).

## 2. Materials and Methods

### 2.1. Patients’ Recruitment

Eighteen (n = 18) HI and twenty-four (n = 24) AA patients were enrolled after dermatological control at the Italian private dermatological clinic, Studio Rinaldi (Milan, Italy). The dermatological control was conducted by Dr. Fabio Rinaldi (HMAP, Human Microbiome Advanced project, Giuliani S.p.A, Milan, Italy, Former Director of the Dermatology Unit at Policlinico Multimedica in Milan), the dermatologist who participated in the study design and enrolment procedure of patients. For each AA patient, essential background data were collected at baseline according to the guidelines of the National Alopecia Areata Foundation [31]. Healthy patients were enrolled following clinical examination and in the absence of any history of dermatological or scalp disorders. Cohorts met the following criteria: (i) no consumption of antibiotics in the last 30 days before the sampling; (ii) no consumption of probiotics in the last 15 days before the sampling; (iii) not pregnant or breastfeeding; (iv) absence of other dermatological diseases; (v) no consumption of anti-tumour or immunosuppressive drugs (patients receiving oral corticosteroids were excluded) and not having undergone radiation therapy in the 3 months before the sampling; and (vi) no topical or hormonal therapy on the scalp in the 3 months before the sampling. Participant recruitment spanned one year, encountering substantial challenges in identifying a sufficient number of individuals for both groups. Meeting the criteria for medical visits and fulfilling the requirements for clinical diagnosis proved to be a demanding task. The time constraints imposed by the research project precluded any extension of the recruitment period. The Severity of Alopecia Tool (SALT) score, an established metric, offers a standardized method for assessing the degree of hair loss in individuals with AA. This score was derived during the clinical examination, wherein the scalp was divided into quadrants, and scores were assigned based on the observed percentage of hair loss in each quadrant. Scores range from 0 to 100, reflecting 0–100% scalp hair loss. The scores were then averaged to obtain the overall severity score, providing a comprehensive assessment of alopecia severity. Only terminal hair, excluding vellus or intermediate hairs, was accounted for in the SALT scoring process.

### 2.2. Nutrient Intake

Enrolled HI and AA patients were asked to fill out a 7-day dietary survey at the time of enrolment, being instructed by a dietician on how to record the food and beverages consumed. The food surveys were analysed by Winfood software (Winfood 2.7, Medimatica S.r.l, Colonnella, Italy) to estimate the energy intake and the percentage of macro- and micro-nutrients. Data collected were compared with the tables of food consumption and recommended dietary intakes of the Italian National Institute of Nutrition and Food Composition Database in Italy [32].

### 2.3. Samples Collection

Each subject collected a pre-prandial faecal sample in the morning on three different days of the same week. After collection, samples were immediately mixed with RNA *later* (Thermo Fisher Scientific, Waltham, MA, USA) (ca. 5 g, 1:2 [wt/vol]) under anaerobic conditions (AnaeroGen, Oxoid Ltd., Basingstoke, UK). Suspended samples were stored at −80 °C until further analyses. Urine samples were also collected in the same day, immediately frozen to avoid bacterial overgrowth, and stored at −20 °C until metabolomics analysis.

### 2.4. 16S rRNA Gene Sequencing

Faecal samples were subjected to total DNA extraction using the Spin Kit for Soil (MP Biomedicals, Milan, Italy) according to the manufacturer’s instructions. To analyse the bacteria, specific primers targeting the V3-V4 region of the 16S rRNA gene (*Escherichia coli* position 341–805; forward 341 F: CCTACGGGNGGCWGCAG and reverse 806R: GACTACNVGGGTWTCTAATCC) were used. The amplicons were cleaned (Agencourt AMPure kit, Beckman Coulter, Brea, CA, USA), and DNA was quantified (Quant-iT PicoGreen dsDNA kit, Invitrogen, Waltham, MA, USA). The quality and purity of the library were checked using a high sensitivity DNA kit (Agilent, Palo Alto, CA, USA) by the Bioanalyzer 2100 (Agilent). The library was prepared, and pair-end sequencing was carried out at the Genomic Platform of Fondazione Edmund Mach in Italy, using the Illumina MiSeq system (Illumina, San Diego, CA, USA). The raw paired-end FASTQ files were demultiplexed using idemp (https://github.com/yhwu/idemp/blob/master/idemp.cpp, accessed on 4 March 2022) and imported into Quantitative Insights into Microbial Ecology (Qiime2, version 2018.2). The sequences were quality filtered, trimmed, de-noised, and merged using DADA2 pipeline. Chimeric sequences were removed through the consensus method in DADA2. A taxonomy classifier was trained on the Silva database using r132 reference sequences, trimmed to the V3-V4 region of 16S rRNA gene, and applied to paired-end sequence reads to generate an operational taxonomic unit (OTU) table.

### 2.5. Faecal and Urine Volatile Organic Compounds (VOCs)

A sensitive assay to identify VOCs was used to evaluate the metabolic features in faecal and urine samples obtained from total forty-two HI and AA patients. In order to obtain the best extraction efficiency, the micro-extraction procedure was performed as described in [33] with slight modifications. Two grams faecal or urine samples with addition of 5 μL of 4-methyl-2-pentanol (final concentration 0.3 mg/L) were placed into 20 mL glass vials and sealed with polytetrafluoroethylene (PTFE)-coated silicone rubber septa (20 mm diameter; Supelco, Bellefonte, PA, USA). The samples were then equilibrated for 30 min at 60 °C. At the end of sample equilibration, a conditioned 50/30 μm DVB/CAR/PDMS fibre (Supelco) was exposed to headspace for 50 min to extract volatile compounds by CombiPAL system injector autosampler (CTC Analytics, Zwingen, Switzerland). VOCs were thermally desorbed by immediately transferring the fibre into the heated injection port (220 °C) of a Clarus 680 (Perkin Elmer, Beaconsfield, UK) gas chromatography machine equipped with an Rtx-WAX column (30 m × 0.25 mm i.d., 0.25 μm film thickness) (Restek, Shanghai, China) and coupled to a Clarus SQ8MS (Perkin Elmer) with source and transfer line temperatures kept at 250 and 210 °C, respectively. The injection was carried out in splitless mode for two minutes, and helium was used as the carrier gas at flow rate of 1 mL/min. The oven temperature was initially set at 35 °C for 8 min, then increased to 60 °C at 4 °C/min, to 160 °C at 6 °C/min, and finally to 200 °C at 20 °C/min and held for 15 min. Electron ionization masses were recorded at 70 eV in the mass-to-charge ratio interval, which was *m*/*z* 34 to 350. The GC-MS generated a chromatogram with peaks representing individual compounds. Each chromatogram was analysed for peak identification using the National Institute of Standard and Technology 2008 (NIST) library. A peak area threshold of >1,000,000 and 90% or greater probability of matches was used for VOCs identification, followed by the manual visual inspection of the fragment patterns when required. To quantify the identified compounds, the internal standard area was used by interpolation with the area of each compound.

### 2.6. Statistical Analyses

All statistical analyses were performed in R programming version 4.1.1. Statistical analyses of the microbiome composition of patients were carried out using “phyloseq” and “microbiome” R packages [34]. Data were rarefied to even sequencing depth. Alpha diversity (Chao1 and Shannon indexes) was computed using relative abundances and fitted on a linear model that included pathology, age range, and their interactions as the main effect variables. The model met the assumptions of the analysis of variance (ANOVA). Two-way ANOVA and post hoc Tukey’s HSD test were further used to determine in depth differences in alpha diversity indexes of microbial communities across different groups (pathology, age range, their interactions). Beta diversity was calculated on centred log-ratio-transformed (CLR transformation) relative abundance data, and ordination was plotted using principal component analysis (PCA), considering the compositional nature of the microbiome data. The permutational multivariate analysis of variance (PERMANOVA) on beta diversity measures was used to compare the groups age range, gender, and pathology and explain any differences in their microbiomes. The core microbiome was defined as the set of OTUs present in at least 50% of the samples. The core microbiome was identified using the function “core_microbiome()” in the “microbiome” package in R. Differentially abundant taxa in pathology status (AA vs. HI) were estimated by two different methods. In the first method, the CLR transformed the microbiome data of all taxa, which were analysed and plotted in a Bland–Altman-style plot using “ggplot” package in R. The second method used linear discriminant analysis (LDA) and LDA effect size (LEfSe) algorithms. The LEfSe algorithm used the Wilcoxon rank-sum test and linear discriminant analysis (LDA) with the cut-off LDA score (log10) set as 2. DIABLO was used to integrate multiple omics datasets (microbiome, nutrition, urine VOC, and faecal VOC). DIABLO was performed using the R package “mixOmics”. This approach is based on a variant of the multivariate statistical technique generalised canonical correlation analysis. Subsequently, a process of adjusting the parameters (performed by the “tune.block.splsda” function) was carried out to find the most suitable number of significant predictors in every dataset to minimize the misclassification rate [35]. The effectiveness of the model was assessed using 10-fold cross-validation. Blocks corresponded to microbiome dataset (CLR-transformed data, aggregated to genus level including the differentially abundant taxa identified before), nutrition dataset, urine VOC, and faecal VOC. The loading weights of each selected variables on each component was represented with plotLoadings function. Power analysis was carried out in R version 4.1.1. using the package “pwr” (https://github.com/heliosdrm/pwr, accessed on 20 November 2023). The analysis parameters were as follows: Cohen’s d/effect size of 0.8; α (significance level) = 0.05; Group 1: n = 24; and Group 2: n = 18.

## 3. Results

### 3.1. Power Analysis, Demographic Data, and Nutrient Intake

The outcome of the power analysis indicates that, under the specified conditions (including disparate group sizes, a significance level of 0.05, and an effect size of 0.8), there is a 70% likelihood that the study will accurately detect a genuine effect. The demographic data of all recruited volunteers (HI and AA patients), including ethnicity, age, gender, AA subtype, and comorbidities are reported in Table 1. The energy intake and macro- and micro-nutrients consumption were evaluated through a food diary for each recruited individual. Data collected agreed with the tables of food consumption of the Italian National Institute of Nutrition and Food Composition Database in Italy. Based on the dietary pattern, no clustering of the two groups (HI and AA patients), or for males and females and age range (30–50 years and over 50 years), were found (Figure S1).

### 3.2. Gut Microbiome of Healthy and AA Patients

**Figure 1:**
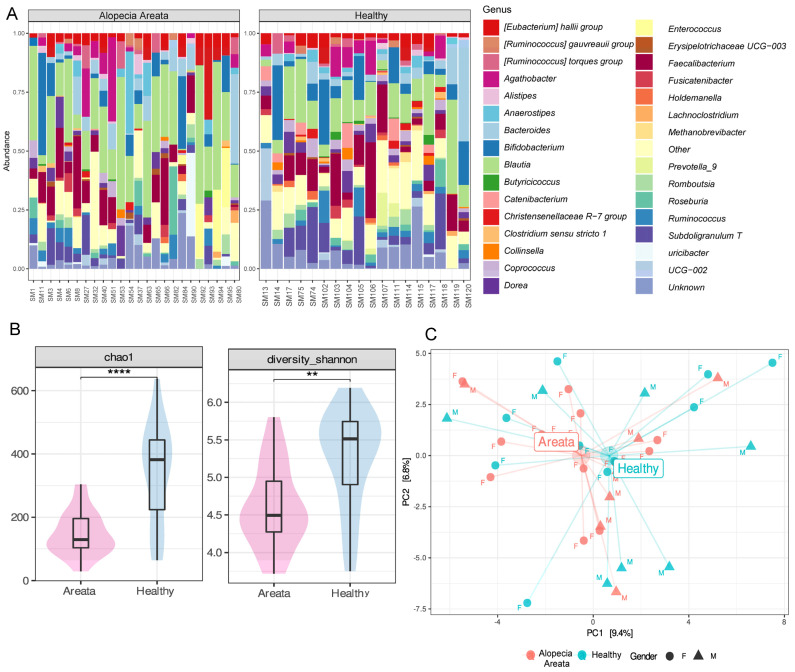
(**A**) Relative abundance at genus level of the faecal bacterial microbiome of healthy individuals (HI) and patients with Alopecia Areata (AA). (**B**) Alpha diversity metrics Chao 1 (species richness, ****, *p* = 0.0001) and Shannon (evenness, **, *p* = 0.001) based on pathology status (AA (pink) and HI (blue)). (**C**) Principal coordinate analysis (PCA) of CLR-transformed microbiome data (beta diversity). Healthy individuals are coloured in blue, while Alopecia Areata patients are denoted with red. Gender is shown using triangles for males and circles for females.

The gut microbiome composition of HI (n = 18) and AA (n = 24) patients was analysed by Illumina MiSeq with an output of total number of 2,500,293 reads, ranging between 78,337 and 153,677 per sample. We identified ten bacterial phyla across the 42 samples combined. However, the relative abundances at phylum level did not show a great variability between HI and AA patients. *Firmicutes* was the most abundant phylum (81.64%) followed by *Actinobacteriota* (8.49%) and *Bacteroidota* (7.62%). The relative abundances at the genus level for the two recruited groups (HI and AA patients) showed higher variability, although sometimes with common features (Figure 1A). *Blautia* (20.2%) and *Faecalibacterium* (8.83%) were the most abundant genera in both aggregated microbiomes, followed by *Bifidobacterium* (7.15%) and *Bacteroides* (5.82%). Compared to AA, *Bifidobacterium* and *Subdoligranum* relative abundances were higher in HI samples. To determine the core microbiome at the genus level, bacterial genera present in more than 50% of the samples were identified using various detection thresholds, with the lowest set at 0.01%. This led to the identification of nine principal core genera for AA, which were *Blautia*, *Faecalibacterium*, *Bifidobacterium*, [*Eubacterium*] *halii* group, *Bacteroides*, *Subdoligranum*, *Dorea*, *Agathobacter*, and *Ruminococcus* (Figure S2). These genera were commonly found in both groups, with the first six being the most frequently detected.

Although there was a core microbiome, the abundance of individual bacterial taxa varied greatly across individuals. There was significant variation in alpha diversity, as measured by Chao1 (species richness) and Shannon (evenness) indexes, which was influenced by factors such as pathology, age range, and gender, as determined by a linear model (Chao1: R^2^ = 0.268, *p* = 0.0247; Shannon: R^2^ = 0.226, *p* = 0.046). The analysis of variance showed that pathology was the most significant factor contributing to variations in alpha diversity indexes (Chao1: *p* = 0.0002; Shannon: *p* = 0.0007), explaining 31.6 and 26.2% of the variance, respectively (Supplementary Table S1). Subsequent post hoc comparison carried out on pathology levels (AA vs. HI) showed that patients with Alopecia had significantly reduced species richness (*p* = 0.00001) and evenness (*p* = 0.001) compared to healthy individuals (Figure 1B). Ethnicity, AA subtype, and comorbidities had no variability and were therefore not included in statistical analyses. Finally, correlations between the alpha diversity indexes and SALT score were not considered for further elaboration due to low variability of the data (SALT score) and small sample size (n = 24).

To investigate whether the taxonomic composition of bacteria communities (beta diversity) was influenced by different variables, the count data were subjected to CLR transformation and ordination was computed with PCA. The results of the PCA (Figure 1C) indicated that the centroids of the pathology groups were close to each other and did not significantly impact the overall microbiota composition. This finding was further confirmed by PERMANOVA analysis, which indicated that none of the variables (i.e., pathology, gender, and age range) individually had a significant effect on the microbiota composition, nor did their pairwise interactions (Supplementary Table S2). The only exception was the interaction between gender and age range, which was found to be significant (*p* = 0.03) in relation to the microbiome but explained only 6.86% of the variance (Supplementary Table S2).

**Figure 2:**
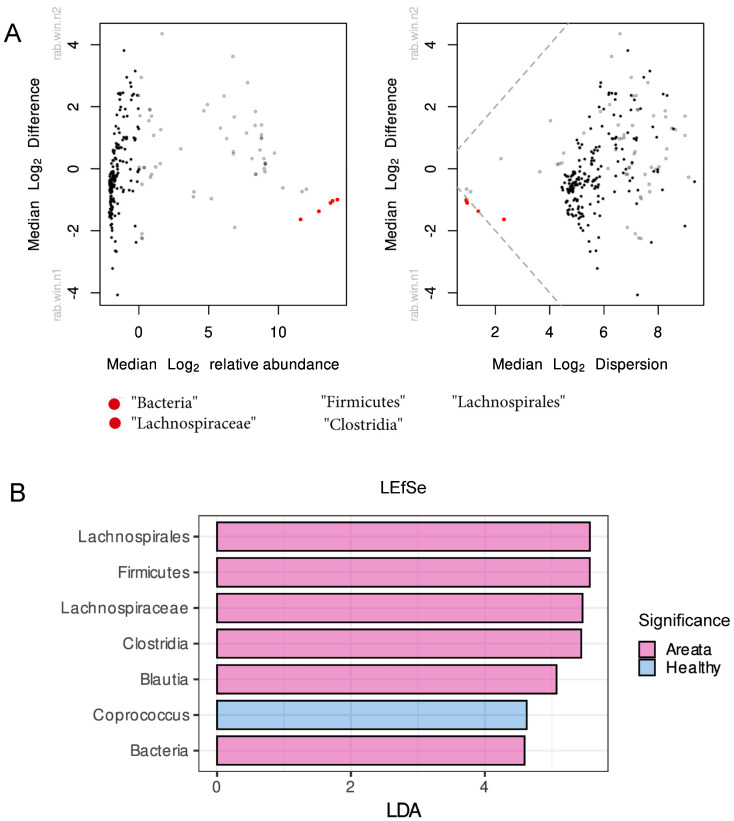
(**A**) Bland–Altman and effect plot of differentially abundant taxa calculated by assessing the fold change of CLR-transformed microbiome counts. Each point on the plot represents a taxonomic feature, colored black if not statistically different between groups, red if identified as significantly different, and grey taxa for which the significance is uncertain. (**B**) Differentially abundant taxa calculated using linear discriminant analysis and LDA effect size algorithm (LEfSe) with cut-off for LDA score set at 2. Pink colour presents the differentially abundant taxa for Alopecia Areata, while with blue colour for the Healthy.

The analysis of differential abundant taxa was carried out using centred log-ratio-transformed relative abundance data across all taxonomic levels and results were plotted on a Bland–Altman-style plot (Figure 2A). The results of the analysis showed that various bacteria taxa, such as *Bacteria*, *Firmicutes*, *Clostridia*, *Lichnospirales*, and *Lachnospiraceae*, were significantly different between pathologies. The results of the LEfSe analysis were largely consistent with the previous method used, with several bacteria taxa including *Lachnospirales*, *Firmicutes*, *Lachnospiraceae*, *Clostridia*, *Blautia*, and *Bacteria* (LDA > 4) identified as biomarkers for AA, while *Coprococcus* (LDA > 4) was identified as a biomarker for HI (Figure 2B).

### 3.3. Integrated Multi-OMICs Analysis on Pathology Regimen (DIABLO)

**Figure 3:**
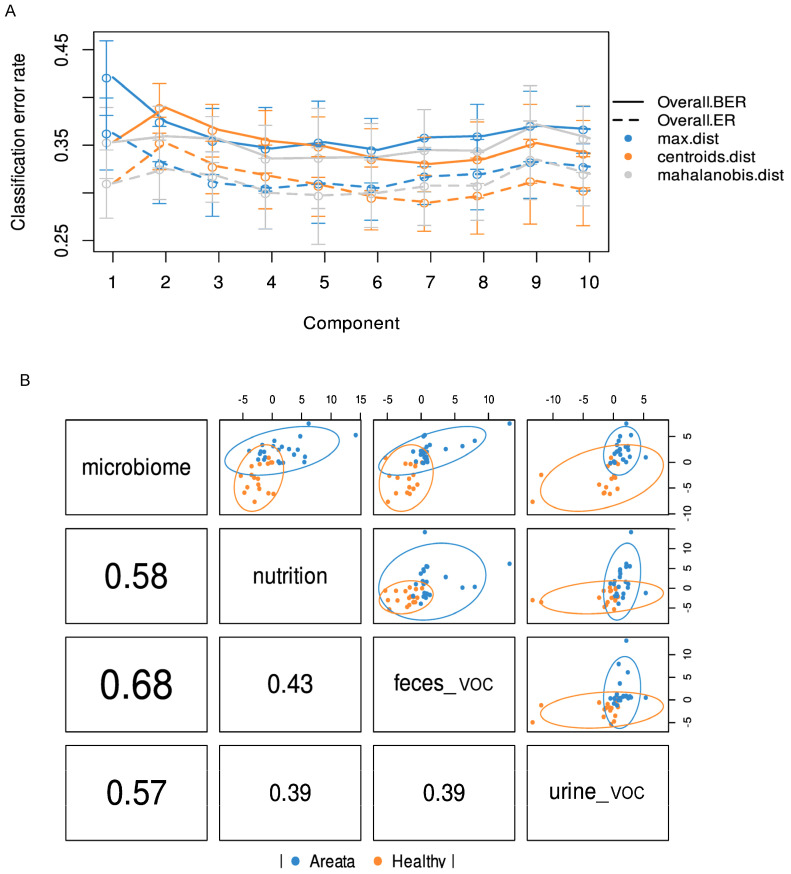
(**A**) Error rates classification based on the partial least square discriminant analysis (PLS-DA) and added components. (**B**) First component correlation results from PLS-DA of multi-omic datasets (nutrition, CLR-transformed microbiome count data, urine VOC, and faecal VOC). Blue circles present the Alopecia Areata datapoints while with orange the Healthy.

**Figure 4:**
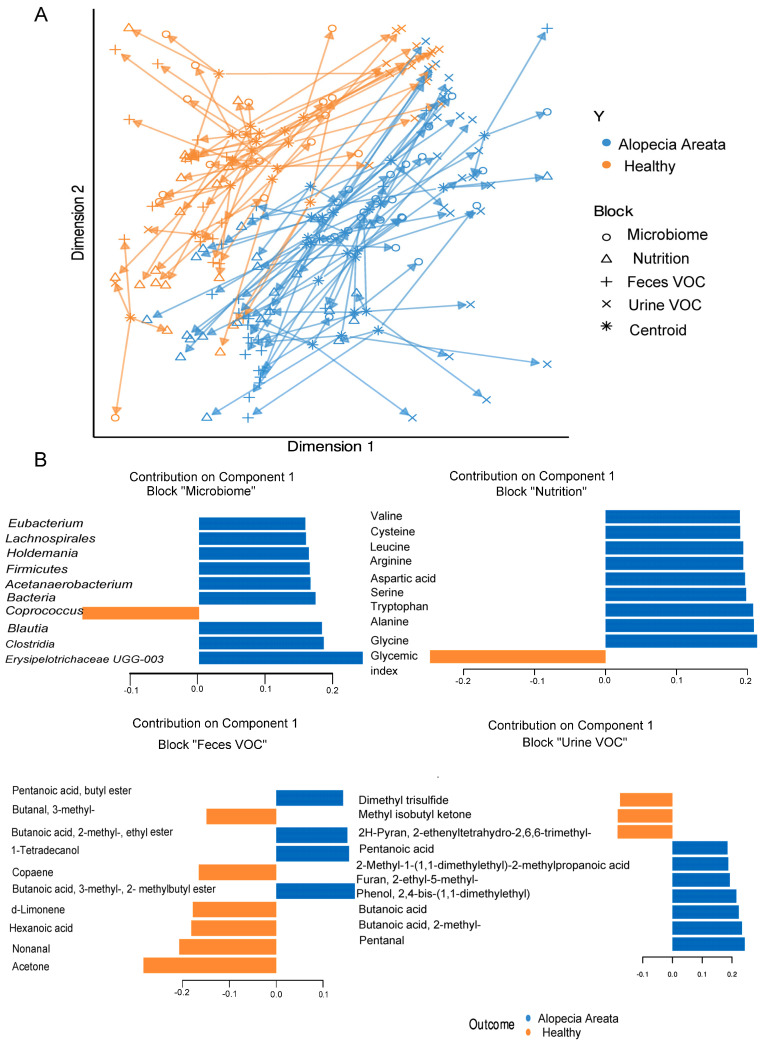
(**A**) Overall ordination of the samples (AA and HI) accounting for both components used in multiblock PLS-DA analysis. (**B**) Top 10 predictors and their loading weights for the first component. Colours indicate the class (pathology) in which the variable has the maximum level of expression. Orange colour denotes healthy individuals (HI), and blue colour denotes Alopecia Areata (AA) patients.

A total of 97 metabolites were found in the urine and 105 in the faecal samples of all recruited individuals, which were further integrated for the supervised multivariate analysis. To assess the interplay across, gut microbiota composition, nutrition, and metabolites (urine and faecal VOC) in relation to the observed impact of pathology, the multi-OMICs datasets were integrated using DIABLO. In the initial analysis, a blocked partial least squares discriminant analysis (PLS-DA) model was constructed using numerous components. The objective was to assess the reduction in the error rate as more components were added, as illustrated in Figure 3A. Error rates decreased somewhat and reached a plateau when two components were used. Hence, the most suitable number of components for blocked PLS-DA analysis was two, for each of the omics datasets. When examining the correlation among the three datasets, the most robust correlation was identified between the microbiome and faecal VOC datasets (Figure 3B). On the contrary, the correlation between urine VOC and nutrition datasets showed the weakest interaction, similar to the relationship observed between urine VOC and faecal VOC, as depicted in Figure 3B. The overall ordination of the samples accounting for all blocks showed that the separation according to pathology status is decent (Figure 4A). Overlapped samples in the middle of the ordination plot resulted in the final error rate of approximately 35% for the multiblock PLS-DA (Figure 4A). The optimally selected key predictors (top 10) of each group included several taxa of the gut microbiota, i.e., *Firmicutes*, *Clostridia*, *Lachnospirales*, *Blautia*, *Holdemania*, *Acetanaerobacterium Erysipelotrichaceae* UCG-003, and *Eubacterium* as biomarkers for AA, while *Coprococcus* for HI. Nutrition predictors for AA included mainly amino acids, i.e., valine, leucine, arginine, serine, and aspartic acid, while the predictor for HI was the glycaemic index. Faecal VOC predictors associated with HI were 3-methylbutanal, copaene, d-limonene, nonanal, and acetone, whereas AA was associated with 1-tetradecanol, butanoic acid, 2-methyl-, ethyl ester and butanoic acid, 3-methyl-, and butyl ester. Finally, urine VOC predictors for AA included pentanal, butanoic acid, pentanoic acid, butanoic acid, 2-methyl-, furan, and phenol-2,4-bis(1,1-dimethylethyl) (Figure 4B). For HI the urine, VOC predictors were dimethyl trisulfide, 2H-Pyran, 2-ethenyltetrahydro-2,6,6-trimethyl, and methyl isobutyl ketone (Figure 4B).

## 4. Discussion

In recent years, there has been increasing interest in studying the impact of genetic and environmental factors on the gut microbiota and its role in human health. Alopecia Areata is a type of autoimmune disease (AID) that is challenging to manage, while the focus of treatment is primarily lying on, controlling its symptoms and associated comorbidities. Recently, some evidence has suggested a relationship between the gut microbiome and AA [23,29,36,37,38]. However, the complex correlations between microbial groups and metabolites that could be responsible for disease development/persistence have not yet been clarified. We studied a cohort of eighteen healthy Italian adults and twenty-four adults affected by AA. The composition of the gut microbiota was examined through sequencing the 16S rRNA bacterial gene using Illumina MiSeq technology. We also performed metabolite analysis of volatile organic compounds using urine and faecal samples.

The microbiota composition on phylum level was concordant with the composition of a healthy human gut microbiome (e.g., *Firmicutes*, *Actinobacteriota*, and *Bacteroidota*) [39]. An imbalance in the abundance of such naturally occurring taxa could result in “gut dysbiosis” [40], but that was not observed in our case. Since the intestinal microbiota on the phylum level was considered relatively stable over time, the core microbiome was established at genus level, which consisted by *Blautia*, *Faecalibacterium*, *Bifidobacterium*, [*Eubacterium*] *halii* group, *Bacteroides*, *Subdoligranum*, *Dorea*, *Agathobacter*, and *Ruminococcus* for both AA and HI. The presence of these genera has been documented in several large metagenomic studies which tried to establish a common adult healthy gut microbiome [41,42,43]. The composition of the gut microbiome did not exhibit significant differences between groups. However, when considering the intra-individual variability, as measured by alpha diversity metrics, notable distinctions were found, particularly in relation to factors such as age range, gender, and pathology status. This aligns with previous research findings [44,45,46]. Pathology status (AA vs. HI) was the most significant factor explaining 20–30% of variance, with significantly reduced species richness and evenness for AA individuals.

Decreased alpha diversity has been repeatedly found in the microbiota composition of patients with AIDs, such as rheumatoid arthritis [47], but also recently in AA patients [48]. Regardless, beta diversity showed no differences as described in several prior studies [23,29,49]. Differentially abundant taxa analysis cumulatively showed that Firmicutes, *Lachnospirales*, *Lachnospiraceae*, *Clostridia*, and *Blautia* were biomarkers for AA, while *Coprococcus* was a biomarker for HI. Within the *Lachnospiraceae* family, *Blautia* is a genus that was previously identified as biomarker of AA with increased abundances compared to the HI [23,38]. Further, *Blautia* has been found to be associated with other disorders such as epilepsy [50], schizophrenia [51], Hashimoto [52], and irritable bowel syndrome, which indicates the high possibility that it is a marker of an “imbalance” in the gut [53]. Imbalances in the gut microbiota can influence intestinal permeability (leaky gut) and even though evidence is scarce, there is a strong link between the induction and progression of AA and gastrointestinal disorders (IBS-like symptoms) [54]. On the other hand, under our experimental conditions, *Coprococcus* was the sole biomarker for HI. *Coprococcus* is known for its capacity to produce butyrate, and the combined literature on *Coprococcus* and the gut microbiota–brain axis points towards enhanced butyrate production and the reduced colonisation of pathogenic clades as factors explaining its association with health effects [55]. Its reduced prevalence has also been associated with AA [23] and has been correlated with reduced amounts of beneficial SCFAs and increased pro-inflammatory proteins [28].

To deepen the investigation of relevant biomarkers, DIABLO was used. DIABLO is a method of discriminant analysis that reduces the dimensionality of multivariate data. It was developed to identify highly correlated and biologically relevant signatures from different OMICs techniques [35]. The key predictors relevant to the microbiome dataset were similar to those found by differential abundant analysis, with the exception of *Holdemania*, *Acetanaerobacterium*, *Eybacterium*, and *Erysipelotrichaceae* UCG-003 among the ones related to AA. The family of *Erysipelotrichaceae* and the species of *Holdemania* were previously found as differentially abundant taxa for AA patients [29]. *Eubacterium* is a major butyrate and propionate producer in the gut and its species, like *E. hallii* and *E. rectale*, have been widely associated with beneficial health effects. However, defining the genus *Eubacterium* can be a complex task, since a number of species originally categorized under this genus have since been reclassified into either pre-existing or new genera [56], possibly affecting the accuracy of taxonomic assignment by the marker gene. The genus *Acetanaerobacterium* is not very well documented in gut microbiome studies. Nevertheless, high prevalence of *Acetanaerobacterium* has been associated with type 2 diabetes [57] and prostate cancer patients [58]. Even though DIABLO is meant to identify coherent patterns between datasets that change with respect to different phenotypes with high accuracy and biological relevance compared to other methods, this model requires test validation [35]. Due to our small sample size, data could not be split and used as training set for the validation. Small sampling sizes are sometimes unavoidable in clinical research, and in our case, it was due to unsuccessful recruitment within a time period of one year and further time constrains to complete the project. Studies conducted with small sample sizes have their limitations but also their advantages when interpreted with caution [59,60]. Acknowledging the fact, we used three different approaches for the microbiome data interpretation. The concordance of microbiome biomarkers between DIABLO and the other methods partially validates the model’s accuracy of prediction. Another limitation of the study could be ascribed to the amplicon-based metagenomics analysis targeting 16S rRNA region. Although this is the most widely used technique to reveal the complexity of human gut microbial consortium, an inherent limitation of the amplicon sequencing method is its compositional information in terms of the relative abundances of the individual members of the community (OTUs, amplicon sequence variants [ASVs], and taxa) [61]. To limit this effect, we conducted all microbiome data analyses based on a compositional data processing workflow [62].

The gut microbiota and associated microbial metabolites can be altered by both diet and host physiology. Diet acts as a key factor of this bidirectional relationship, which can either promote the growth of beneficial bacteria or contribute to the proliferation of potentially harmful microbes [63]. The gut microbiota, through metabolite production, modulates signalling pathways involved in the homeostasis of intestinal mucosa. Through DIABLO analyses, the esters of branched chain fatty acids (BCFAs) (butanoic acid, 2-methyl-, ethyl ester and butanoic acid, 3-methyl-, butyl ester) and branched chain amino acids (BCAA) (i.e., valine, leucine) were identified as predictors of AA subjects in faecal VOC and the nutrition dataset, respectively. It is known that BCFAs are mainly produced during the fermentation of BCAA (valine, leucine, and isoleucine) by the intestinal microbiota [64]. BCFAs have been proposed as markers of colonic protein fermentation, a process that leads to the concomitant production of other protein fermentation products such as ammonia, phenol, *p*-cresol, or biogenic amines; molecules that can cause cell damage on the intestinal environment [65]. High levels of 3-methylbutanoic acid in faeces have been related to human depression and cortisol levels, and more recently, the role of BCFA on the regulation of glucose and lipid metabolism has been also examined [64]. An increased production of BCFA esters in AA could be also referred to oxidative stress, which promotes lipid peroxidation downstream. More precisely, the production of alcohols and carboxylic acids in the presence of oxygen-reactive species (radical-mediated lipid peroxidation) leads to esters formation [66]. Oxidative stress (OS), an unbalance between the oxidation and antioxidant defence systems, is believed to be associated with AA and may trigger the collapse of hair follicle-immune privilege [67].

Bacterial metabolites might modulate immune responses [68], like SCFAs (butanoic and pentanoic acid) and BCAA (butanoic acid, 2-methyl), which were identified as markers of urine VOC in AA. The biochemical interpretation of these results is hampered by the difficulty of assessing the origin of SCFAs and BCAAs in urine due to the possibility of their generation by microbial activity in other parts of the body. However, SCFAs interact with G-protein-coupled receptors (i.e., GPR43) and modulate inflammation by reducing proinflammatory cytokines (i.e., TNFα and IFNγ) and increasing the production of anti-inflammatory cytokines (e.g., IL-10). Nonetheless, a previous work showed an association between the SCFA urinary profiles and the disease status of patients with ulcerative colitis, where higher butyrate levels in patients at remission were observed, and it was suggested that the urinary output could reflect the levels of butyrate produced in the gut [69]. The increase in pentanoic and butanoic acid in AA patients (urine samples) found in our study could be attributed to homeostasis.

Further, we found phenol-2,4-bis(1,1-dimethylethyl)(2,4-DTBP) and furan as urine predictors in AA subjects. 2,4-DTBP is a major component of essential oils and can be obtained from various groups of organisms (e.g., bacteria, plants, fungi, flowering plants) [70], and it exhibits antioxidant properties and demonstrates potential as an anticancer, antifungal, and antibacterial agent [71]. Moreover, 2,4-DTBP is a synthetic antioxidant used in polyethylene cross-polymer (PEX) water distribution pipes and food-related plastics as a valuable stabilizer preventing material degradation and disintegration. Notably, 2,4-DTBP utilized for the durability and endurance of plastics can leach from these materials and has been found in breast milk, cord blood, and placental tissue [72]. Interestingly, 2,4-DTBP has been implicated in causing depigmentation after occupational exposure and has resulted in a variety of systemic abnormalities, including thyroid, liver, and/or splenic changes after exposure. Based on these observations, they suggested that 2,4-DTBP may be capable of inducing inflammation in organs beyond the skin [73]. In a prior investigation, a robust association was established between detectable urinary furan levels and γ-glutamyltranspeptidase (γ-GT), a recognized marker for liver damage. The presence of furan in a wide array of heat-processed foods has raised significant concern, given its classification as a “possible carcinogenic compound to humans”. Given furan’s genotoxic nature, elevated exposure levels may signify a health risk [74]. Therefore, further exploration is essential to understanding metabolic pathways and the potential toxicity of dietary furan in humans.

## 5. Conclusions

In this study, DIABLO was used to identify microbial and metabolite biomarkers associated with Alopecia Areata (AA). The metabolome predictors highlighted potential bioactive intestinal and microbial metabolites linked to host disease aetiology. The evaluation of faecal and urinary volatile organic compounds (VOCs) suggested a non-invasive approach for identifying proinflammatory metabolites associated with low-grade inflammation [75], including AA. While the gut microbiome composition aligned with previous studies, microbiome biomarkers between AA and healthy individuals were consistent across three analysis methods. However, the limitations of such as a small sample size hindered data splitting for model validation. Despite this, the concordance of microbiome biomarkers partly validated their predictive accuracy. Amplicon-based metagenomics analysis targeted the 16S rRNA region, and while widely used, has limitations in providing compositional information about the relative abundances of individual community members. We addressed this using a compositional data workflow (log-ratio transformation) [62]. This study lays groundwork for future investigations into disease pathogenic mechanisms, including exploring specific genera’s roles in AA onset and the interaction between AA patients’ metabolomes and their immune systems. However, our approach does not definitively establish causality between changes in microbiota and metabolome and AA development or progression, necessitating validation experiments with individuals affected by AA from symptom onset.

## Figures and Tables

**Table 1:** Demographic data of all volunteers including gender, age, ethnicity, Severity of Alopecia Tool (SALT) score, and comorbidities.

Demographic Data	AA	HI
	n = 24	n = 18
Men n, (%)	8 (33.33%)	7 (38.88%)
Women n, (%)	16 (66.67%)	11 (61.12%)
Age (y, mean ± DS)	40.00 ± 11.76	45.00 ± 9.86
Ethnicity (n, %)		
White	24 (100%)	18 (100%)
Salt Score (mean ± DS)	87.46 ± 1.87	n.a. *
AA subtype (mean ± DS)		n.a.
Alopecia areata	24 (100%)	
Comorbidities *		n.a.
Celiac disease	2 (8.33%)	
Allergic rhinitis	1 (4.16%)	

* n.a. = not applicable. In the case of healthy individuals (HI), these data were not registered.

## Data Availability

The datasets presented in this study can be found in online repositories. The names of the repository/repositories and accession number(s) are as follows: NCBI BioProject—PRJNA857562 (https://www.ncbi.nlm.nih.gov/bioproject/?term=PRJNA857562+, accessed on 11 July 2022).

## Supplementary Materials

The following supporting information can be downloaded at: https://www.mdpi.com/article/10.3390/nu16060858/s1. Figure S1: Pseudo-heat map showing nutritional intake, macro- and micronutrients of healthy individuals (HI, n = 18), and Alopecia Areata (AA, n = 24) patients. Cluster analysis was carried out by correlation and linkage criterion of the average distance for both clustering rows and columns; Figure S2: Core microbiome at the genus level of bacteria present in more than 50% of the samples detected in various abundance levels with the lowest set at 0.01%. Core microbiome was determined for 18 healthy individuals (HI) and 24 Alopecia Areata (AA) patients; Table S1: Analysis of variance (ANOVA) significant terms and explained alpha diversity indexes variance (%); Table S2: Permutational multivariate ANOVA (PERMANOVA) and significant variables and their R2 (%) on microbiome data from 18 healthy individuals (HI) and 24 Alopecia Areata (AA) patients.
