# Supplementary material for: Exploring the Gut Microbiome and Metabolome in Individuals with Alopecia Areata Disease
Source: Nutrients. 2024 Mar 15;16(6):858. doi: 10.3390/nu16060858 (PMC10975414; doi:10.3390/nu16060858)
Supplement: Supplementary file 1 [file nutrients-16-00858-s001.zip › nutrients-2873521-supplementary.pdf]

FIG S1

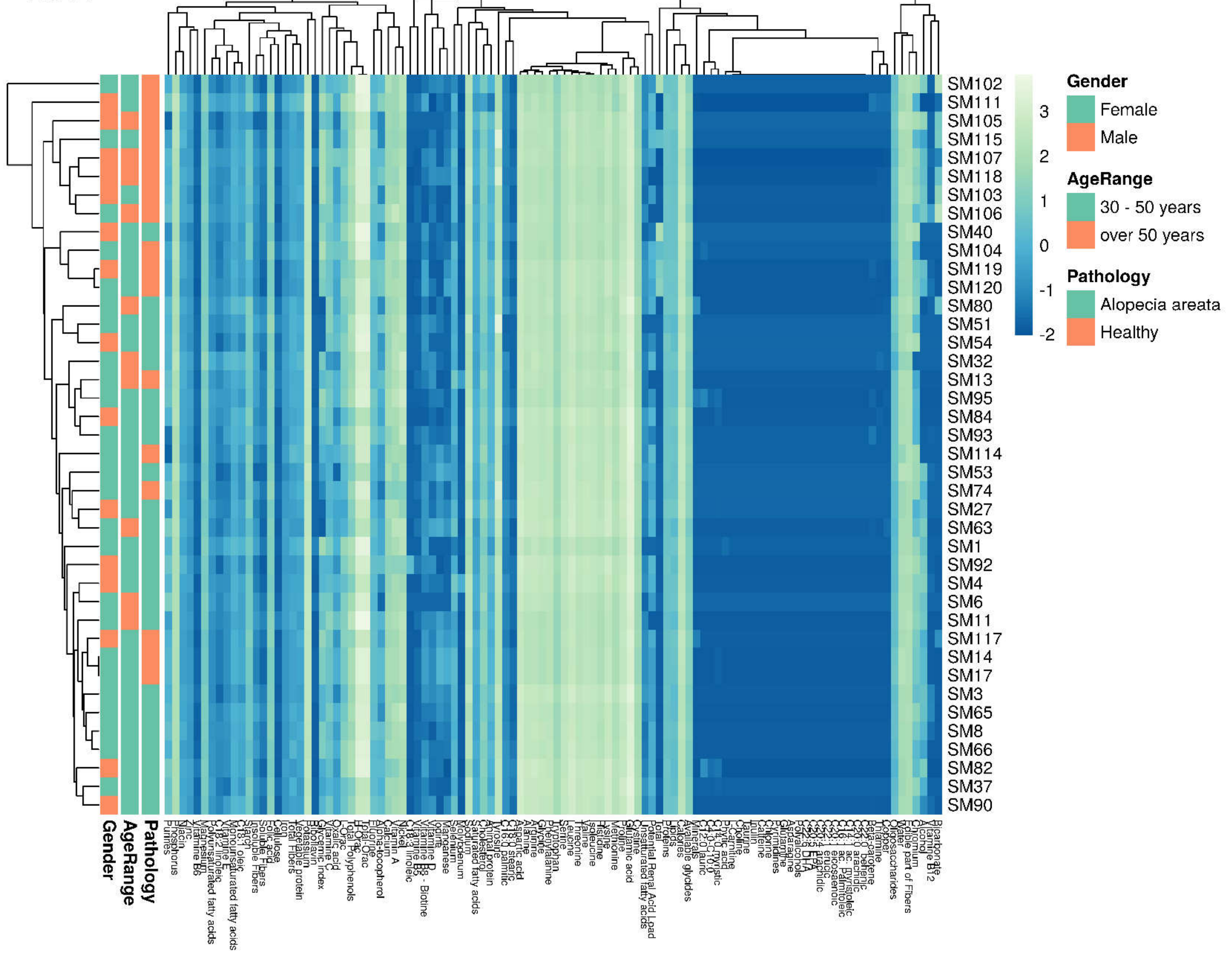

Figure S1. Pseudo-heat map showing nutritional intake, macro- and micronutrients of healthy individuals (HI, n=18) and Alopecia Aerata (AA, n=24) patients. Cluster analysis was done by correlation and linkage criterion the average distance, for both clustering rows and columns.

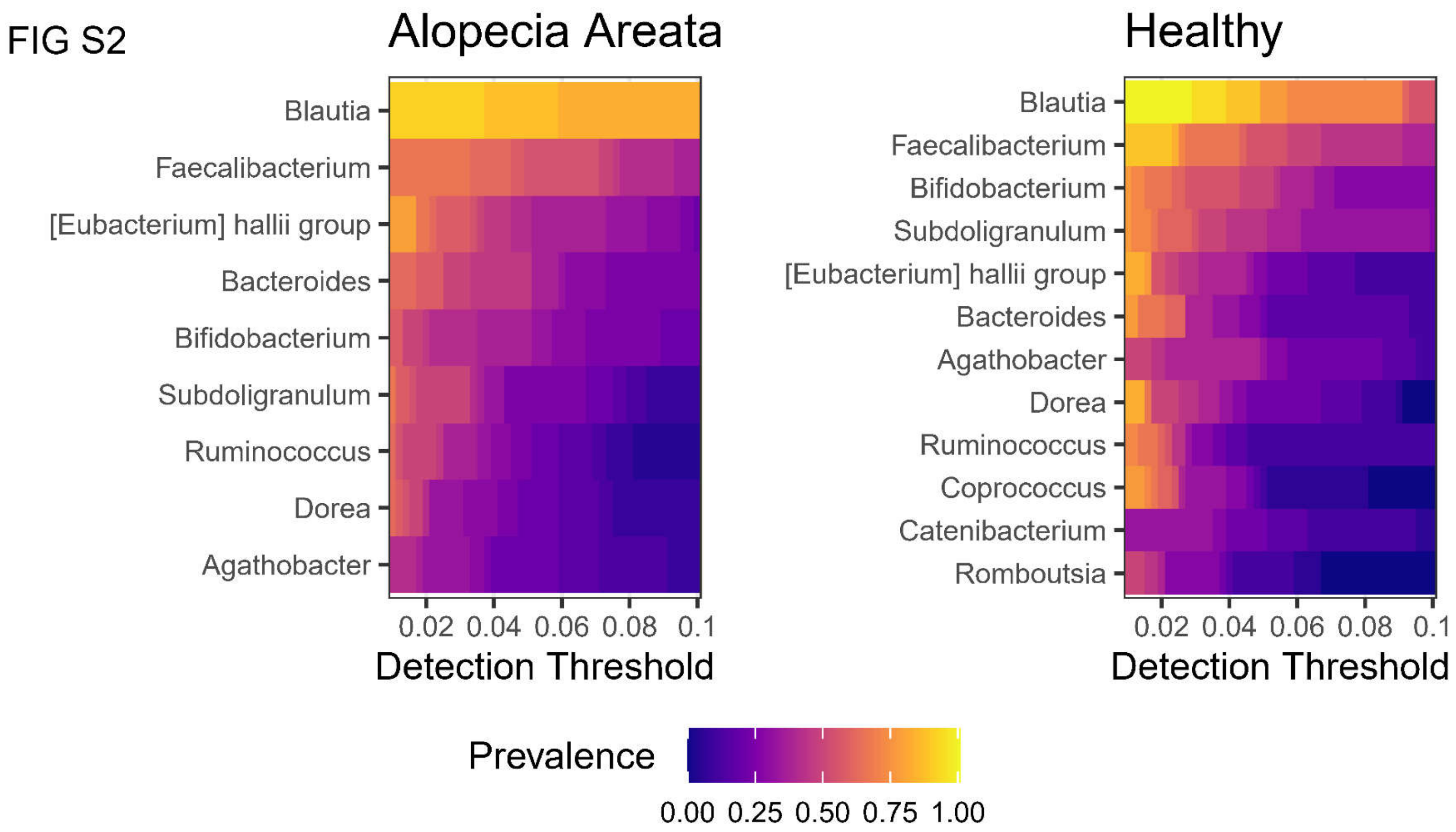

Figure S2. Core microbiome at the genus level of bacteria present in more than 50% of the samples detected in various abundance levels with the lowest set at 0.01%. Core microbiome was determined for 18 Healthy individuals (HI) and 24 Alopecia Areata (AA) patients.

Table S1. Analysis of variance (ANOVA) significant terms and explained alpha diversity indexes variance (%).

Diversity Index	Variables	Pr(>F)	Variance Explained %
Chao1	AgeRange	0.931	0.256
Chao1	Gender	0.0895	5.49
Chao1	Pathology	0.000203	31.6
Chao1	AgeRange:Gender	0.758	0.998
Chao1	AgeRange:Pathology	0.199	6.09
Chao1	Gender:Pathology	0.759	0.171
Chao1	AgeRange:Gender:Pathology	0.976	0.00161
Chao1	Residuals	NA	55.4
Shannon	AgeRange	0.67	1.53
Shannon	Gender	0.137	4.41
Shannon	Pathology	0.000775	26.2
Shannon	AgeRange:Gender	0.707	1.32
Shannon	AgeRange:Pathology	0.14	7.92
Shannon	Gender:Pathology	0.962	0.0044
Shannon	AgeRange:Gender:Pathology	0.921	0.0191
Shannon	Residuals	NA	58.6

Table S2. Permutational multivariate ANOVA (PERMANOVA), significant variables and their R^2 (%) on microbiome data from 18 healthy individuals (HI) and 24 Alopecia Aerata (AA) patients.

**Variables**	**Pr(>F)**	**R^2**
Pathology	0.06	3.39
Gender	0.73	2.40
AgeRange	0.06	6.39
Pathology:Gender	0.69	2.43
Pathology:AgeRange	0.13	6.33
Gender:AgeRange	0.03	6.86
Residual	NA	72.21
Total	NA	100.00
